# Factors influencing nurses’ pain assessment and management of road traffic casualties: a qualitative study at a military hospital in Ghana

**DOI:** 10.1186/s12873-024-01016-8

**Published:** 2024-06-18

**Authors:** Thomas Kwame Tata, Lillian Akorfa Ohene, Gladys Akorfa Dzansi, Lydia Aziato

**Affiliations:** 1 37 Military Hospital, Neghelli Barracks Liberation Rd 37, Accra, Ghana; 2 Department of Public Health Nursing, School of Nursing and Midwifery, University of Ghana, P. O Box LG 43, Legon, Accra, Ghana; 3 Department of Adult Health Nursing, School of Nursing and Midwifery of Ghana, P. O Box LG 43, Legon, Accra, Ghana; 4 University of Health and Allied Sciences, PMB 31, Volta Region, Ho, Ghana

**Keywords:** Trauma, Emergency unit, Pain assessment, Nursing, Ghana

## Abstract

**Background:**

Evidence shows that patients who visit the surgical and trauma emergency units may be discharged with untreated or increased pain levels. This study explored nurses’ pain assessment and management approaches at a trauma-surgical emergency unit in Ghana.

**Methods:**

Seventeen nurses who work in the trauma department participated in this qualitative exploratory descriptive study. In-depth individual interviews were conducted, and the thematic analysis was utilized to identify emerging themes and subthemes.

**Results:**

Three main themes were identified: patient pain indicators, pain management, and institutional factors influencing pain management. The study revealed that nurses rely on verbal expressions, non-verbal cues, physiological changes, and the severity of pain communicated. The findings highlighted staff shortage, inadequate resources, and lack of standardized guidelines as factors affecting pain and management.

**Conclusions:**

Although the study offers critical new perspectives on nurses’ experiences regarding pain related issues at the trauma-surgical emergency units, its small sample size limited its generalizability.

## Background

Pain dominates presenting clinical symptoms in patients who visit the emergency unit for treatment, with the prevalence of mild to severe pain reported in the majority [1]. Pain induced by Road Traffic Injuries (RTIs) dominates the causes of impairment and fatality globally [2]. It is projected to be the third leading factor to induce disability-adjusted life years lost [3]. Globally, about 50 million people are injured yearly through Road Traffic Accidents (RTA), and the burden of RTIs internationally has been growing. It is projected that RTIs will become the fifth leading cause of mortality by 2030 if no interventions are implemented [4]. Ghana has also been experiencing a rise in RTAs, contributing to a growing burden on healthcare systems [5]. In 2017, the Motor Traffic and Transport Department (MTTD) of the Ghana Police Service indicated that 20,444 vehicles were involved in RTAs, with 3,300 pedestrian knockdowns and 12,166 travelers injured [6]. All these casualties who sustain various injuries would likely end up in hospitals for medical care, in which pain management is inescapable. Consequently, it is crucial to understand and improve pain assessment and management practices for RTA casualties, as pain can significantly impact their well-being and recovery.

Pain assessment accuracy is important for effective management [7]. The quest for pain relief has been attributed to casualties’ emergency department visits for medical interventions [8]. Effective pain management depends on the knowledge and skills of the nurse to accurately assess the pain and make an appropriate clinical decision. Despite extensive research and updated guidelines on pain management, satisfying patient expectations for adequate and timely pain relief remains a challenge in most emergency departments [9]. According to Wheeler et al. [9], nurses have limited knowledge in assessing and managing pain in the emergency department. It was also identified that nurses in Ghana undermine the pain experience of patients, which makes nurses neglect or inadequately administer the prescribed analgesics [10]. This reiterates that adequate assessment of the pain of RTA casualties by nurses who are mainly at the frontline of triaging would result in prudent management and good pain relief [10].

Various factors can influence nurses’ pain assessment and management approaches for RTA casualties. Cultural and contextual factors significantly shape patients’ perceptions and expressions of pain [10]. In Ghana, cultural beliefs influence how individuals experience and communicate pain, impacting how nurses assess and manage pain in this population [10]. The availability of resources and the healthcare system in hospitals also affect pain assessment and management practices. In resource-limited settings like Ghana, pain medications and specialized equipment may be limited and challenging, which influences effective pain management [11]. Additionally, hospital organizational policies and protocols can influence pain assessment and management approaches in nurses’ practice. Nurses’ knowledge and attitudes toward pain may greatly influence their assessment and management practices.

Adequate training and education on pain management enable nurses to adopt evidence-based practices, while inadequate knowledge and misconceptions can lead to suboptimal pain management [12]. Workload and time constraints also impact nurses’ ability to assess and manage pain effectively. In busy hospital settings, nurses may have limited time to devote to each patient, hindering their ability to accurately assess pain levels and develop comprehensive pain management plans. Effective communication and collaboration among healthcare professionals are vital for optimal pain assessment and management. Collaborative efforts among nurses, physicians, and other healthcare providers ensure comprehensive pain management strategies are implemented [13].

Evidence shows that patients may be discharged with untreated or increased pain levels [8, 13, 14]. This study explores and analyzes factors influencing nurses’ pain assessment and management of RTA casualties in a military hospital in Ghana. This will ultimately contribute to developing evidence-based guidelines and interventions for improved pain assessment and management in this population.

## Method

### Study objectives

The two objectives of this study are to (a) to explore nurses’ pain assessment and management approaches at a trauma-surgical emergency unit at a Military setting in Ghana; (b) to explore institutional factors influencing nurses’ assessment and pain management of RTA casualties at a trauma-surgical emergency unit at a Military setting in Ghana.

### Study design

The study adopted an exploratory, descriptive qualitative approach. A careful selection of this approach and meticulous application of its tenets to clinical studies offers opportunities for robust, in-depth, and rich empirical evidence [15]. According to Moen and Middelthon, exploratory designs offer flexible methods to aid the discovery of the perspective of which much is not known [16]. The flexibility nature of the design motivated its application in this study.

### Setting

Military hospitals in Ghana often primarily deal with trauma cases, including RTA casualties [17]. This study was conducted at one of the oldest Military Hospitals of the Ghana Armed Forces in the capital city of Accra, Ghana. The hospital has a bed capacity 1500 with a 38-bed capacity for the trauma surgical emergency department. Nurses in the trauma unit work alongside physicians, surgeons, anesthesiologists, and other healthcare professionals in a multidisciplinary team to provide comprehensive trauma care [18]. Typical of this setting is the high patient volumes due to the nature of emergency care and the prevalence of RTAs. The influx of patients can place a considerable workload on nurses, potentially impacting their ability to dedicate sufficient time and resources to pain assessment and management [19]. However, there seems to be inadequate nursing staff strength at the trauma unit.

### Population

Nurses from the study population were recruited based on inclusion and exclusion criteria. The inclusion criteria for this study included nurses who worked at the trauma surgical emergency unit for at least one year, and they must be at post for the three-month period of data collection and consent to participate in the study. Nurses on any leave (study, sick maternity, or particular military assignment) were excluded. Nurses who were waiting to start their annual vacation within the data collection period were also excluded during the data collection.

### Sampling and recruitment techniques

The study’s participants were purposively selected. The researchers obtained permission from the hospital authorities to access the recruitment site. After receiving authorization from the chief nurse, the first author responsible for the data collection visited the staff during morning meetings and explained the goal and purpose of the research. The inclusion criteria, which included experience of one year’s practice at the unit, were communicated to all potential participants. Nurses who were fit and willing to participate in the research were enrolled after getting their consent before interviews.

### Data collection

Data collection was mainly through qualitative, in-depth individual interviews. Participants were scheduled for face-to-face individual interviews at their own convenience time and place. All the interviews took place at different locations within the hospital. Semi-structured interviews were conducted with the aid of an interview guide. The research objective and research questions informed the content of the interview guide. Some questions included “*Share your pain assessment practice on RTA casualties*” and “*What factors influence patient pain assessment and management at your unit?*” All the interviews were conducted in English. Probing questions were asked to explore the topic in-depth. The interviews lasted between 40 and 73 min. The interviews were audio recorded and transcribed with the participant’s permission for further analysis.

### Data analysis

A thematic data analysis approach was adopted to identify, analyze, and interpret the patterns in the data [20, 21]. Data collection and analysis occurred iteratively; thus, after each interview, the recorded audio was played several times, which enabled immersion in the data. Emerging concepts guided the subsequent interviews. The transcripts were also read several times to familiarize with the data. A line-by-line coding was conducted on all transcripts to identify concepts. Codes identified during the initial coding were grouped and labeled. Code grouping, labeling, and relabeling continued until clear distinctions were made between clusters of codes, which became themes. The relationships and links in themes brought about the determination of subthemes.

### Trustworthiness

The study applied the principles of rigor, including credibility, transferability, dependability, and confirmability [21]. The strategies encompassed strict adherence to the study protocols to enhance the credibility of the methodology and findings. Prolonged engagement was employed, where participants were allowed to articulate their responses to inquiries and express their experiences. In addition to doing member checking, the researchers provided the participants with the findings to validate the accuracy of their ideas captured. The transparency about the participants and the research setting ensured transferability. For dependability, the study provides detailed descriptions of interpretations and replies while maintaining an audit trail that documents all the techniques utilized. The confirmability process allowed us to engage the participants during the data analysis. This checked researcher biases and ensured that the findings resonated with the participants.

### Ethical considerations

The researchers obtained ethical approval from two institutions: the Institutional Review Board of the University of Ghana at the Noguchi Memorial Institute for Medical Research (NMIMR-IRB CPN 021/18–19) and 37 Military Hospital (37MH-IRB IPN/260/2018). An agreement was obtained from the hospital management, the hospitals’ gatekeepers. Every individual involved in the study provided their signature on a permission form after fully understanding the contents of the information sheet and seeking clarification through inquiries as needed. The participants were informed about the presence of a clinical psychologist during the conversation. All participants gave consent to participate in the study. During the interview, nurses who experienced emotional distress were allowed to articulate and express their feelings openly.

## Results

### Demographic characteristics

A total of fifteen nurses participated in the study. Seven were males and the rest eight females. Eight of them were military nurses and seven were civilian nurses. The participants age ranged between 28- 45years and all of them had tertiary level of education in nursing. The participants included a mix of nursing ranks; thus, seven nursing officers, four staff nurses, and three Senior Nursing officers with one principal nursing officer. The participant’s years of nursing experience ranged between 4–22 years and years of experience at the Trauma Surgical Unit was between 1 and 9 years.

### Thematic results

The analysis of the seventeen interviews identified three themes, underpinned by nine descriptive subthemes (Table 1). The main themes include “*patient pain indicators, pain management, and institutional factors influencing pain management”.* The subsequent sections describe and discuss the themes subthemes.

**Table 1:** Themes and subtheme of nurses and pain assessment of casualties

Main Themes	Subthemes
Patient Pain indicators	• Verbal expressions • Non-verbal cues • Physiological changes • Severity of pain
Pain management	• Pain experiences • Non-pharmacological • Pharmacological
Institutional factors in patients’ pain management	• Staffing • Pain managing resources

## Patient Pain indicators

### Verbal expressions

One crucial strategy the nurses use in pain assessment is patients’ expression and description of pain. All seventeen participants in this study reported that verbal expressions are first assessed in conscious patients. Nurses said they ask patients about the presence of pain, location, severity, and nature of the pain. According to the participants, in cases where patients are overwhelmed with severe pain, they may make peculiar sounds such as crying, moaning, wailing, or even shouting to express unbearable pain.

*Very often, we ask whether there is pain, and in most* cases, *you may hear them moaning, shouting, and they may say to you that they are in severe pain.* Some *can say which part of the body the pain is. For those who sustained injuries, either the head, neck, arm, or leg***N5**.

*Some of these injured patients will constantly cry out indicating that they are in severe pain. They wail and groan; crying and shouting out so loud to draw nurses’ attention to their pain***N2**.

### Non-verbal cues

According to participants in this study, non-verbal signs are instrumental in casualty pain assessment. Non-verbal signs are assessed for accident victims who are conscious but are unable to verbalize their pain. Non-verbal pain expressions are also present in unconscious patients. Typically, frowning facial expressions, clenching teeth, and guarding the site of pain are signs nurses look out for. Some participants also mentioned that patients’ general body composure and lack of interest in their immediate surroundings are other signs of severe generalized body pain. Others also said that such patients mostly show restlessness and forceful and extreme pressure in their grasp on the attending nurse or medical staff.

*Injured patients squeeze their faces when in pain****N4.***

Some close *their eyes tight and clench their teeth****N9.***

*I know that they often guard the pain site and even avoid certain maneuvers****N7***.

### Physiological changes

Participants in this study were aware of some physiological changes that occur in the human body when casualties experience pain. Mainly, the participants mentioned abnormal vital signs reading as the first change to assess. Profuse sweating was also mentioned as an indicator of pain. Some said a loss of appetite and fluid retention can be observed.

*In injured patients, high pulse and blood pressure* could *indicate pain****N2***.

*Some patients profusely sweat when there is pain, and their respiration reading is high****N13***.

### Severity of pain

Assessing the severity of pain was deemed as one key factor for effective pain management. Evidence from this study revealed that nurses at this study site recognized that the patient’s description of the severity of pain was the most critical factor in pain management. However, they further acknowledged using pain rating scales, which were not adopted in their facility. One participant explained that some health professionals speculate about patients faking the severity of their pains. It was also evident that participants’ perceptions of pain influenced their judgments regarding the interpretation of patients’ pain reports.

*We depend greatly on the patients’ judgment of the severity of their pain****N9***.

*Because some patients have foreknowledge and want some particular medication, they may rate their pain as severe. But through facial expressions and the behaviors, you may know that the pain is not that severe as the patient might claim even though pain is subjective****N5.***

*I know of the zero to ten rating scale. But there is no such rating scale* here. We *need to adapt one which will be a factor to influence pain management****N3***.

### Pain management

#### Personal pain experiences

The findings from this study revealed that medical staff’s personal experiences and perceptions of pain influenced their approach to patients’ pain management. All the participants in this study confirmed that they had experienced different types of pain in the past. Participants’ pain types range from muscle sprain to backache, post-operative pain, and canular site pain.

*Personally, I know how painful an injury can be. I once had a dislocation and cut during a football game. It was excruciating****N3***.

*Well, I’m constantly having back* pain, *and it can be severe when I attempt to lift patient****N6***.

*I had surgery two years ago, and the post-operative pain was very unbearable****N11.***

It was mentioned that personal experiences influenced perceptions regarding how staff interpreted patients’ pain. According to participants in this study, the sudden nature of injuries among their clients was enough to confirm the reality of pain. However, some of the participants reported that colleagues from different cultural backgrounds can sometimes be adamant about patients’ continued reports of pain. There were perceptions that some patients exaggerated pain.

*I also used to think that some* patients *exaggerate pain, but with what I have experienced in the past, I no longer think that way. However, some colleagues still do based on their own perceptions about pain or their cultural background****N6***.

It was obvious that medical staff pain experiences were an important influencing factor in the pain management of casualties.

*Sometimes, I recall how patients with similar injuries in the past expressed their pain and how they responded to the interventions. I* often *use* such *knowledge to guide my current situation****N8.***

*My own pain experience helped me understand my patient’s complaint of pain****N3***.

#### Pharmacological

According to participants in this study, pain management in road traffic accident victims is of great concern to the patients and the medical staff. All thirteen participants in this study alluded to using pharmacological agents as the first measure to managing casualties’ pain. The emerging medications from the data include non-steroidal anti-inflammatory drugs (NSAIDs), antipyretic and mild and strong opioids.

*The common drugs we use here include* paracetamol, *diclofenac, tramadol, pethidine, and dihydrocodeine****N5.***

Participants were also quick to mention that they sometimes use a combination of drugs to achieve sedation and relaxation in patients with severe pains.

*In severe cases, we* combined paracetamol *with pethidine or diclofenac with tramadol, which works effectively for most patients****N13***.

*We combined Midazolam with diclofenac to relax the apprehensive patients who are scared****N11***.

The route of pain drug administration was another factor the participants considered necessary. Most respondents mentioned intramuscular and intravenous routes as the most effective means to achieve a quick positive response to pain administration in injured patients.

*Mostly, the drugs are given intramuscularly. For example, we give diclofenac, tramadol, and pethidine through the intramuscular; however, if the pain is severe, we give the pethidine intravenous for the patient to have a quick relief****N3***.

Some participants cautioned against the frequent and quick use of pain medications. The commonly cited reasons included medication addiction, and in some cases, the pain relief may mask significant symptoms for diagnosis.

*Hmmm, I also know that pain medication such as opioids is not given in patients with acute abdominal pain. This is to* avoid masking *judgment during clacking. The physician must complete the initial assessment before such* medications *are given for pain****N10***.

#### Non-pharmacological

This study identified three main non-pharmacological strategies for relieving pain. Most of the participants cited immobilization, positioning, and patient reassurance. Traditionally, in injured patients, fractured limbs are splinted to immobilize the part. The cervical collar, cuff, and collar were other items for immobilizing the cervical spine and clavicular fractures. These non-pharmacological procedures help to reduce pain and prevent further complications. Positioning techniques were other non-pharmacological measures the participants outlined. These involve using bed accessories such as pillows, sandbags, heart tables, paper boxes, and many more to support injured patients in comfortable positions to relieve pain.

*When you immobilize the injured part, the patient is comfortable. It is fundamental but effective when you immobilize the affected part****N4***.

Positioning *is also* an *integral part of RTA pain management; the patient will often call for* assistance *to change position to get some pain relief”****N11.***

The participants also acknowledged the power of patient reassurance as a means of pain relief. According to them, reassuring injured persons helped to calm them, increased their trust in the medical staff, and raised their hopes. Reassurance to patients was also deemed necessary in all circumstances, even when pain medication was given. When you talk to them, it is a form of distraction, a diversional therapy to take their minds off pain.

*Hmmm, reassurance sometimes works like magic; that is, just talking to them to calm them, they feel fine and hopeful that someone cares about their pain* N4.

*In* accident *cases, talking to them alone doesn’t work. We give them pain medication first before we follow up to reassure them”**** N1.***

### Institutional factors in patients’ pain management

#### Staffing

Most participants outlined staff shortage as an institutional leading factor that affects pain assessment and management. According to participants in this study, the patient to nurse ratio at the emergency department was always high. Thus, it may be as high as one nurse to forty casualties during peak seasons whereby some casualties may even be nursed in wheelchairs. The consequences include increased workload for nurses, poor patient monitoring and assessment, and frequent sick-off by the staff.

*We have always complained about our staff strength****N10***.

*We basically and mostly work understaffed****N1***.

*Assessing the effects of* drugs *given* for *pain is the best, but we don’t have enough professionals to monitor and evaluate our casualties****N9***.

Another dimension of the staff and institutional-related factors was the nurses’ knowledge of pain assessment and management. Most participants confirmed that at the initial stage of employment, they had some basic understanding of pain management. However, they expected their employers to provide avenues for additional training on pain management. Some participants recalled having some training with the in-service coordination unit in the past. Others never had such opportunities for training on pain management.

*Yes, we were all taught pain management in training, but you do not expect that knowledge to apply still. New ways of pain management are emerging, and we need that continuous training. Our institution must enable ongoing training in that regard ****N2***.

#### Pain managing resources

This study identified several resources to facilitate pain assessment, management, and evaluation. These include pain management protocol, equipment and logistics, and working space. The participants narrated a lack of standardized protocol for managing pain at the emergency unit, which makes work more complicated during mass casualties. Participants lamented inadequate beds, stretchers, and medical supplies, such as pain medications and examination gloves. It was also reported that physical space at the emergency department was an impediment and a source of stress in pain assessment and the overall management of patients. According to the participants, the space of the emergency department was too small, limited, and often overcrowded.

*There is no standard guideline or written protocol for pain management in this hospital; I suggest that there should be a written* protocol *that will back nurses’ actions in pain management****N7***.

*Our major problems are logistics, inaccessible drugs, no bed, and no stretcher, among several other needs****N10***.

*Sometimes, there are many patients* here, *but the place is too small to move freely between them. This place is simply congested****N11.***

## Discussion

Two research objectives guided this study. Regarding objective one which explored nurses’ pain assessment and management approaches at a trauma-surgical emergency unit, the findings revealed that nurses rely on verbal expressions, non-verbal cues, physiological changes, and the severity of pain communicated. The second objective explored institutional factors influencing nurses’ assessment and pain management of RTA casualties at a trauma-surgical emergency in Ghana; and the findings also highlighted staff shortage, inadequate resources, and lack of standardized guidelines as factors affecting pain and management.

Typically, patients’ verbal cues gave professionals insight into the casualty’s pain. In a related study by Booker and Haedtke [22], verbalization of pain was reported by patients in acute pain situations, including RTA casualties. Pain can be verbalized through crying, shouting, screaming for help, moaning, and reporting the presence of pain. Self-reports of the presence of pain have been considered among the most accurate and reliable means of assessing the presence and intensity of pain [23]. This indicates that nurses listening to verbal reports of pain in this study played an important role in the pain assessment of RTA casualties.

Another form of pain assessment reported in this study was looking out for nonverbal cues. These findings conform to some earlier studies that have also shown similar ways of pain expression [22, 24, 25]. Frowning facial expressions, clinching teeth, guarding the site of pain, patients’ general body composure, lack of interest in their immediate surroundings, and restlessness are some of the nonverbal means by which nurses observe pain experiences. Mostly, the nurses used observation of facial expressions as the primary nonverbal expression of pain in RTA casualties. This conforms to the validation of facial expressions of pain reported in previous studies [26–28]. This also suggests that the human face is conspicuous and depicts important messages when attention is paid to it [29, 30]. Although facial pain assessment has been established to have clinical value, especially for non-verbal communicating patients [27], its reliability may be limited by differences in individual facial features such as the shape of the face and hair distribution on the face and scalp. The individual variations in appearance may lead to significant outcomes for facial assessment [31]. This challenges nurses to have adequate knowledge about the various distinctions in facial expression to rely on as a pain assessment tool. However, this study noted that the nurses do not use pain assessment tools to elicit pain and its intensity. It is, therefore, important to use appropriate pain assessment tools to serve as a framework that would guide pain assessment in such vulnerable casualties [32–34].

The participants indicated physiological changes in the human body when casualties experience pain. These included abnormal vital signs reading, profuse sweating, sudden loss of appetite, and fluid retention. Some previous studies have reported a similar positive relationship between acute pain and heart rate [35] and blood pressure [36]. On the contrary, other earlier studies reported that an increase in physiological changes, such as vital signs, may not signify the presence of pain but can suggest the need for pain assessment [37]. For instance, an increased heart rate in an RTA casualty may indicate a physiologic response to excessive haemorrhage. Although some participants of this study indicated awareness of physiological changes in response to the presence of pain, participants mainly used verbal and nonverbal expressions of pain.

The study found that the personal pain experiences of nurses affected the assessment and management of the pain of RTA casualties. The nurses indicated that their previous pain experience provided a better understanding of the patient’s pain experiences, facilitating effective pain intervention. This does not imply that nurses without pain experience cannot effectively manage pain. The skills of pain management can be acquired through learning. It is, however, essential to individualize the pain management of casualties to achieve effective outcomes, as pain is subjective, and generalizing casualties’ pain management may be ineffective [38].

The nurses used both pharmacological and nonpharmacological means for the pain management of RTA casualties. The knowledge of the use of different forms of pain management for RTA casualties conforms to the clinical practice guidelines of the American Pain Society (APS) recommendation that emphasizes a multimodal pain approach [13]. The study found the use of various types of pharmacological drugs in managing the pain of RTA casualties. This aligns with earlier studies outlining various types of analgesia used to manage traumatic injury pains [39]and [40]. The study revealed that apart from the extensive use of NSAIDs and acetaminophen, the nurses knew the use of opioids to alleviate severe pain in RTA casualties. It was, however, limited to the use of only pethidine and tramadol as the only available types among the numerous types recommended by opioid analgesia for traumatic injuries protocol [41].

Nonpharmacological means of pain intervention adopted by the nurses included immobilization, positioning, and reassurance. The nurses immobilized fractured limbs as adjuvant to pain intervention. Arm slings, splints, cardboards, soft bands, and gauze bandages are used to immobilize fractured limbs to prevent further injury, allow adequate blood supply to the area, and help reduce pain [42]. This study saw the positioning of casualties in bed for pain reduction. This supports some studies that indicated pain relief by adopting some positions [43, 44]. The nurses supported the administration of analgesics with reassurance to reduce anxiety and allay fears of casualties. Moseley & Butler found that educating patients on the biological cause of pain contributes immensely to reducing pain [45].

This study also showed an inadequate number of nurses in the emergency unit to take care of the pain of road traffic accident casualties. Recent studies have emphasized the importance of the availability of nurses in accelerating pain relief in patients [46, 47]. Staffing in this context borders on the availability of the right quantity and quality of nursing staff to offer timely pain management services to casualties. Poor staffing leads to rationing of care, which may aggravate the pain, extend hospital stay, and increase the risk of death [47]. Enough nurses at the emergency unit reduce the workload of nurses and enable them to have time to re-evaluate the intervention given to casualties [48]. It also reduces work stress. The nurses indicated receiving no training in pain assessment and management of RTA casualties for the period working at the emergency unit. Training empowers nurses with the requisite knowledge and skills and makes them aware of the various pain assessment tools and appropriate use [34 − 33]. Inadequate training implies that the workforce would lack up-to-date knowledge, skills, and abilities to manage pain among causalities effectively [49]. Therefore, periodic pain assessment and management training for emergency nurses is recommended to enhance effective pain management.

The study revealed a lack of standardized protocol for managing pain at the emergency unit, which makes work more complicated during mass casualties. It identified inadequate beds, stretchers, and medical supplies, such as pain medications, examination gloves, etc. The availability of these materials and equipment facilitates the management of pain among accident casualties [40–42]. Limited physical space at the emergency department was an impediment and a source of stress in pain assessment and the overall management of patients. A decent working environment is necessary, particularly in emergency units of hospitals, to help avoid overcrowding, injuries, and unnecessary delays in the bid to deliver nursing services [48] and [47].

## Implications of the study

This study’s implications significantly shed light on nurses’ challenges in assessing and managing pain in trauma-surgical emergency units. The findings suggest that nurses need more training and education on pain assessment and management in these settings. This is particularly important in resource-limited settings where there may be a staff shortage and inadequate resources. The study also emphasizes the significance of adopting standardized pain evaluation and management protocols to promote consistency in care delivery. This can help to enhance patient outcomes and lower the risk of adverse events caused by ineffective pain management. The study also highlights the importance of more efficient use of available resources in trauma-surgical ERs. This includes providing more staffing resources, ensuring adequate access to medications and other pain management interventions, and developing strategies to mitigate resource constraints and shortages. Overall, this study’s findings have significant implications for nursing practice in trauma surgical emergency units and suggest the need for ongoing efforts to enhance pain management practices and patient outcomes in these settings.

## Limitations

The study only included fifteen participants from one specific hospital in Ghana, so the findings may only apply to some specific emergency units. The findings, therefore, cannot be generalized. Also, the study relied on self-reported participant data, so there may be a possibility for recall bias. There may be cultural biases, where the cultural biases of the researchers or participants may impact the data analysis. For example, the lack of new techniques and procedures for pain assessments and management in a low-resource context could influence participants’ responses.

## Conclusion

In conclusion, even though the study offers critical new perspectives on the experiences of the nurses working in trauma-surgical emergency units, the study’s small sample size, the potential for bias, limited generalizability, lack of quantitative data, and potential cultural biases should be considered when interpreting the findings. Future studies should address these limitations to increase the validity and reliability of findings and provide a greater understanding of the barriers and possibilities involved in enhancing the pain assessment and quality of care in trauma-surgical emergency units. The goal should be to develop successful solutions that may be applied to improve the health of both patients and healthcare providers.

## Data Availability

The datasets generated and analyzed during the study are available from the corresponding author upon reasonable request.

## Abbreviations

RTIs: Road Traffic Injuries
RTA: Road Traffic Accidents
MTTD: Motor Traffic and Transport Department
